# Inhibition of Virulence Factors and Biofilm Formation by Wogonin Attenuates Pathogenicity of *Pseudomonas aeruginosa* PAO1 via Targeting *pqs* Quorum-Sensing System

**DOI:** 10.3390/ijms222312699

**Published:** 2021-11-24

**Authors:** Shiwei Wang, Yuqi Feng, Xiaofeng Han, Xinyu Cai, Liu Yang, Chaolan Liu, Lixin Shen

**Affiliations:** 1 Key Laboratory of Resources Biology and Biotechnology in Western China, Ministry of Education, The College of Life Sciences, Northwest University, Xi’an 710069, China; wangsw@nwu.edu.cn (S.W.); 201831864@stumail.nwu.edu.cn (Y.F.); hanxiaofeng@stumail.nwu.edu.cn (X.H.); caixinyu@stumail.nwu.edu.cn (X.C.); yliu@stumail.nwu.edu.cn (L.Y.); 2 Provincial Key Laboratory of Biotechnology of Shaanxi Province, The College of Life Sciences, Northwest University, Xi’an 710069, China; 3 Antibiotics Research and Re-Evaluation Key Laboratory of Sichuan Province, Sichuan Industrial Institute of Antibiotics, Chengdu University, No. 168, Huaguan Road, Chengdu 610052, China; liuchaolan@edu.cdu.cn

**Keywords:** wogonin, *P. aeruginosa*, quorum sensing, virulence factors, biofilm, molecular docking

## Abstract

*Pseudomonas aeruginosa*, an important opportunistic pathogen, is capable of producing various virulence factors and forming biofilm that are regulated by quorum sensing (QS). It is known that targeting virulence factor production and biofilm formation instead of exerting selective pressure on growth such as conventional antibiotics can reduce multidrug resistance in bacteria. Therefore, many quorum-sensing inhibitors (QSIs) have been developed to prevent or treat this bacterial infection. In this study, wogonin, as an active ingredient from *Agrimonia pilosa*, was found to be able to inhibit QS system of *P. aeruginosa* PAO1. Wogonin downregulated the expression of QS-related genes and reduced the production of many virulence factors, such as elastase, pyocyanin, and proteolytic enzyme. In addition, wogonin decreased the extracellular polysaccharide synthesis and inhibited twitching, swimming, and swarming motilities and biofilm formation. The attenuation of pathogenicity in *P. aeruginosa* PAO1 by wogonin application was further validated in vivo by cabbage infection and fruit fly and nematode survival experiments. Further molecular docking analysis, pathogenicity examination of various QS-related mutants, and PQS signal molecule detection revealed that wogonin could interfere with PQS signal molecular synthesis by affecting *pqsA* and *pqsR*. Taken together, the results indicated that wogonin might be used as an anti-QS candidate drug to attenuate the infection caused by *P. aeruginosa*.

## 1. Introduction

It has been known that the mass use of antibiotics increases bacterial resistance [1] and the development of new drugs has obviously slowed down, leading to a decrease in the cure rate and an increase in the mortality rate for bacterial infectious diseases. Novel therapy strategies have been urgently needed for bacterial infection treatment, especially for the antibiotic-resistance pathogens.

*Pseudomonas aeruginosa*, a Gram-negative opportunistic pathogen, is frequently found in the department of respiratory and critical care medicine and can cause acute and chronic infections in immunocompromised or burned patients. During acute infection, *P. aeruginosa* synthesizes adhesion substances such as extracellular polysaccharides for adherence, secretes protease to destroy the structure of host cells for entrance, and produces some virulence factors such as pyocyanin to defend itself against the immune system of host [2]. To initiate chronic infection, *P. aeruginosa* often forms a biofilm, which relates to extracellular polysaccharide and bacterial motility [3]. Biofilm formation is capable of increasing bacterial drug resistance hundreds of times [4].

Virulence factor production and biofilm formation of *P. aeruginosa* are crucial for its pathogenicity. Most of the virulence factor production and biofilm formation of this bacterium are regulated by quorum-sensing (QS) systems [5,6,7]. The QS system is a cell-to-cell communication system firstly discovered in Gram-negative bacteria. Bacteria produce and detect signal molecules to regulate their behavior in a cell density-dependent manner through QS system [8,9]. It is reported that three classic QS systems including *las*, *rhl*, and *pqs* systems are present in *P. aeruginosa*, although *iqs* was previously reported, recent studies showed that this system is still controversial [10]. The QS system of *P. aeruginosa* is a complex hierarchic circuitry. The *las* system consists of LasI/LasR. LasI synthesizes N-(3-oxododecanoyl)-L-homoserine lactone (3-oxo-C_12_-AHL), and this molecule is detected by transcription regulator LasR. LasR/3-oxo-C_12_-AHL complex activates *lasI* and some other genes such as *rhlI*/*rhlR*, the second QS system, when a threshold concentration is reached. RhlI produces N-Butanoyl-L-homoserine lactone (C_4_-HSL), which is detected by transcription regulator RhlR [11]. RhlR/C_4_-HSL complex activates *rhlI* and other genes such as *rhlAB* for rhamnolipid production [12]. In addition, *P. aeruginosa* has the third QS system, *pqs*, which produces signaling molecule 2-heptyl-3-hydroxy-4-quinolone (PQS) by operon *pqsABCDE* and transcription regulator PqsR [13]. *pqs* system regulates the expression of some virulence-related genes by PqsR such as pyocyanin and biofilm formation [14]. Considering the key roles of the QS systems in virulence factor production and biofilm formation of *P. aeruginosa*, QS systems are considered to be ideal targets for screening drugs to treat bacterial infections [15]. Many quorum sensing inhibitors (QSIs) have been developed for the treatment of bacterial infection, such as hordenine [16], 5-hydroxymethylfurfural [17], rhubarb [18], trans-cinnamaldehyde [19], and salicylic acid [19].

In China, traditional Chinese medicinal herbs have been used to treat diseases for thousands of years. Among them, those with the function of “Qing Re Jie Du” are the most frequently used, and “Qing Re Jie Du” means anti-bacteria and anti-inflammation. Therefore, these medicinal herbs are believed to be safe and promising to screen QSIs [20,21,22]. In this study, in order to identify the active substance with QS inhibitory function for the treatment of *P. aeruginosa* infection, we first examined 18 types of traditional Chinese medicinal herbs with the function of “Qing Re Jie Du”. *Agrimonia pilosa* was found to exhibit the optimal QS inhibitory effects. Further investigation suggested that the active ingredient in *A. pilosa* was wogonin, which showed anti-virulence and anti-biofilm effects. Wogonin reduced the production of the virulence factors like pyocyanin, elastase, and Psl exopolysaccharides and inhibited motility and biofilm formation. In addition, it was also found that wogonin attenuated the rot degree of Chinese cabbage (*Brassica pekinensis*) and improved the survival rate in the animal models (*Drosophila melanogaster* and *Caenorhabditis elegans*) after *P. aeruginosa* infection. Molecular docking analysis showed that wogonin had strong ability to bind PqsR protein and the thin-layer chromatography (TLC) results displayed that wogonin reduced the production of PQS. In summary, wogonin could target *pqs* QS system to weaken the pathogenicity of *P. aeruginosa*, indicating that wogonin might be used as an anti-QS candidate drug to attenuate the infection caused by *P. aeruginosa*.

## 2. Results

### 2.1. Inhibition of P. aeruginosa Virulence and Pathogenicity by the Crude Extracts of A. pilosa

**Figure 1:**
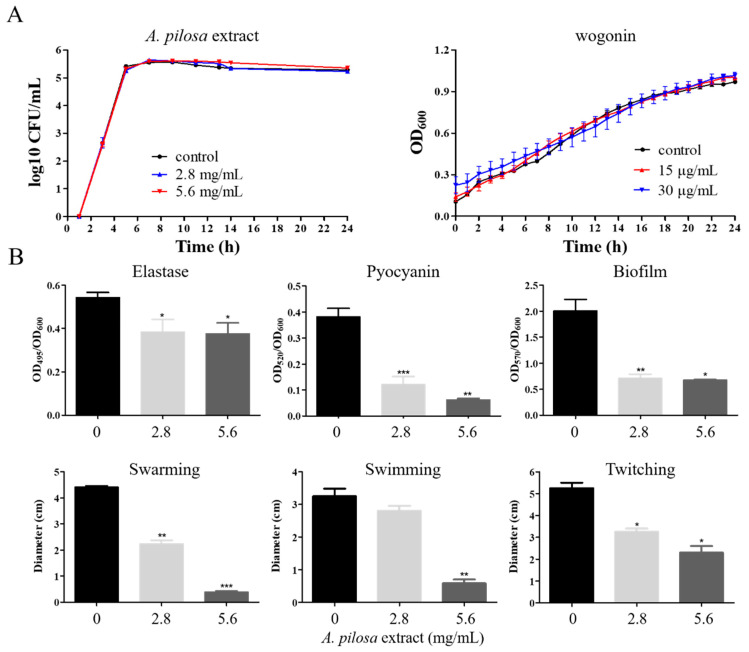
The effects of the crude extracts from *A. pilosa* and wogonin on *P. aeruginosa* PAO1 growth and virulence factors. (**A**) When PAO1 was cultured, various concentrations of the crude extracts from *A. pilosa* and wogonin were used. To examine the effects of crude extracts from *A. pilosa* on growth, colony-forming units (CFUs) were examined every 2 h for 24 h to avoid the interference of the crude extract color. Regarding wogonin, OD_600_ of the culture was measured every 2 h for 24 h. (**B**) The concentrations of the crude extracts from *A. pilosa* used were 0, 2.8, and 5.6 mg/mL, respectively. The change of the phenotypes related to QS systems were examined, including elastase, pyocyanin, biofilm, swarming, swimming, and twitching. The error bars represent standard errors. *, *p* < 0.05; **, *p* < 0.01; ***, *p* < 0.001.

In order to screen potential QSIs of *P. aeruginosa*, 18 kinds of Chinese medicinal herbs with the function of “Qing Re Jie Du” were selected. The active ingredients of them were, respectively, extracted by deionized water and 70% ethanol, and the effects of the crude extracts on the expression of QS-related genes were examined. The results showed that the extracts of *A. pilosa* with deionized water or 70% ethanol both inhibited the most QS-related genes (Table 1) but did not impact the growth of *P. aeruginosa* PAO1 (Figure 1A). Note that the deionized water extracts increased the expression of one QS-related gene, *pqsH*, and inhibited that of the others. However, the 70% ethanol extracts inhibited that of all the tested genes. Therefore, in the following study, 70% ethanol extracts of *A. pilosa* were used. Sd the QS system regulates most of the virulence factors and pathogenicity [7,23], we further examined the effects of the extracts from *A. pilosa* on the virulence phenotypes of PAO1.The virulence-related phenotypes, including motility (twitching, swimming, and swarming), elastase and pyocyanin production, and biofilm formation, were reduced when different concentrations of the 70% ethanol extracts of *A. pilosa* were used (Figure 1B). The pathogenicity detection by the Chinese cabbage model showed that the decay area gradually became small with the increase of the extract concentrations of *A. pilosa* (Figure S1). Therefore, some important component(s) from the extracts of *A. pilosa* would be selected for further study.

### 2.2. Wogonin Inhibited QS-Regulated Genes in P. aeruginosa PAO1

**Figure 2:**
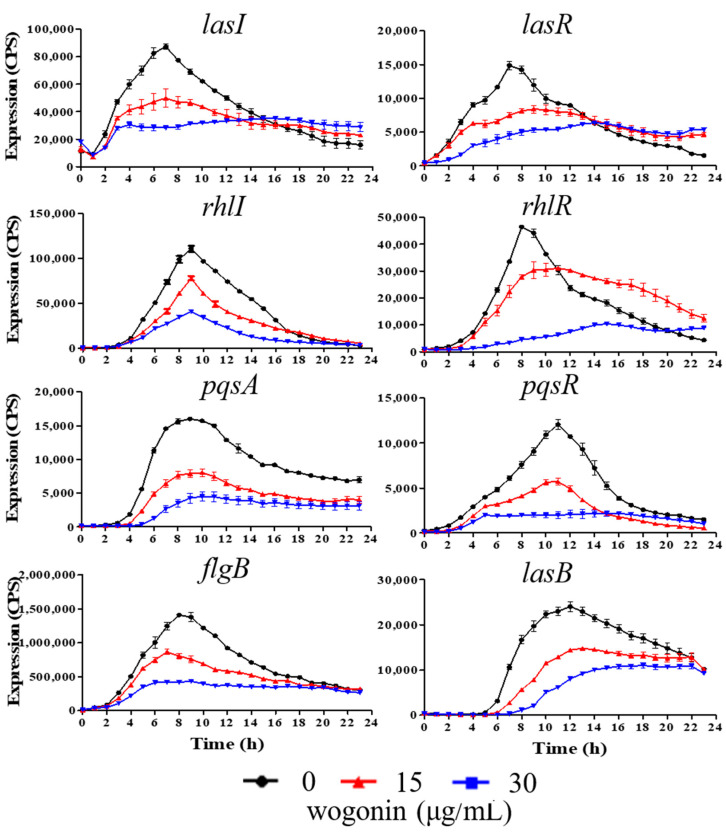
The transcriptional profiles of the genes related to QS systems under the conditions of different concentrations of wogonin. Black lines, no wogonin; red lines, 15 μg/mL wogonin; blue lines, 30 μg/mL wogonin. The promoter activities were measured using a *lux*-reporter system. The relative expression values of various genes were presented as CPS (Count Per Second) normalized to OD_600._ The error bars represent standard errors.

Flavonoids are the most main components of *A. pilosa*, and wogonin as one family of flavonoids has been used to suppress inflammatory responses [24]. We speculated that wogonin might be involved in the anti-QS activity of *A. pilosa*. To examine our speculation, the effects of wogonin on the expression of QS-related genes were first detected. The results showed that 15 μg/mL and 30 μg/mL wogonin inhibited the expression of five genes including *rhlI, pqsA*, *pqsR*, *flgG*, and *lasB* during a 24-h period and reduced the expression of three genes *lasI*, *lasR*, and *rhlR* before ~12 h but not after 12 h (Figure 2). In addition, 15 μg/mL and 30 μg/mL wogonin had no significant effects on PAO1 growth (Figure 1A). The results were similar to those of the crude extracts of *A. pilosa*, indicating that the active ingredient observed in the above-mentioned phenomena might be wogonin.

### 2.3. Inhibition of P. aeruginosa Virulence and Pathogenicity by Wogonin

**Figure 3:**
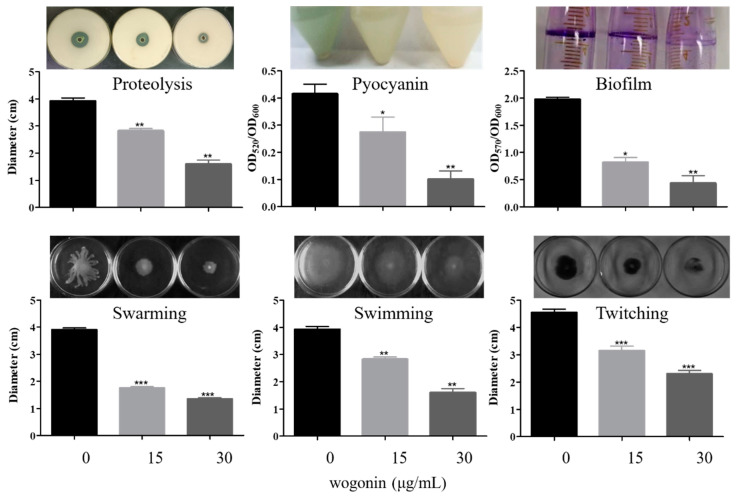
The effects of wogonin on *P. aeruginosa* PAO1 virulence factors, including proteolysis, pyocyanin, biofilm, swarming, swimming, and twitching. The wogonin concentrations used were 0, 15, and 30 μg/mL, respectively. The error bars represent standard errors. *, *p* < 0.05; **, *p* < 0.01; ***, *p* < 0.001.

**Figure 4:**
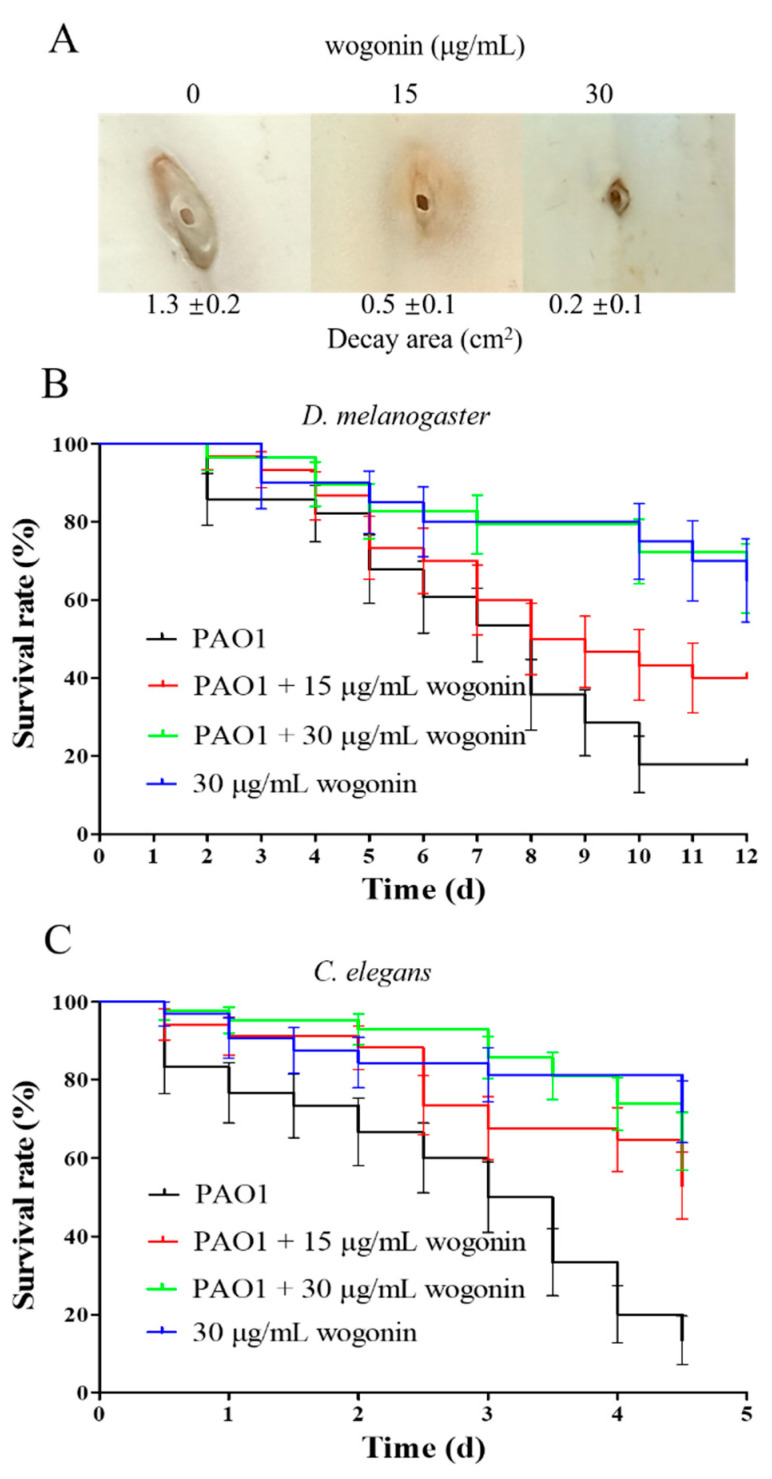
The effects of wogonin on *P. aeruginosa* PAO1 pathogenicity. After infection, the decay area of cabbage (**A**) and the survival rates of *D. melanogaster* (**B**) and *C. elegans* (**C**) were, respectively, measured with or without wogonin addition. Dark lines, only PAO1 infection; red lines, PAO1 infection and 15 μg/mL wogonin addition; green lines, PAO1 infection and 30 μg/mL wogonin addition; blue, only 30 μg/mL wogonin addition. The error bars represent standard errors.

The effects of wogonin on virulence and pathogenicity of *P. aeruginosa* PAO1 were further investigated. The results showed that 15 μg/mL and 30 μg/mL wogonin inhibited motility including swarming, swimming, and twitching, reduced the production of Psl polysaccharide, pyocyanin, and elastase, and impacted the ability of proteolysis and biofilm formation (Figure 3). Interestingly, the concentration of wogonin at 15 μg/mL almost eradicated the 10-h biofilm (Figure S2). Three models in vivo—Chinese cabbage, fruit fly, and nematode—were used to examine the effects of wogonin on the pathogenicity of PAO1. The results showed that wogonin could weaken the rot degree of the stem in the Chinese cabbage infection model and improved the survival rates in the infection models of fruit fly and nematode (Figure 4). The results were also comparable with those of the crude extracts of *A. pilosa*.

### 2.4. Wogonin Mainly Affected the pqs System of P. aeruginosa PAO1

**Figure 5:**
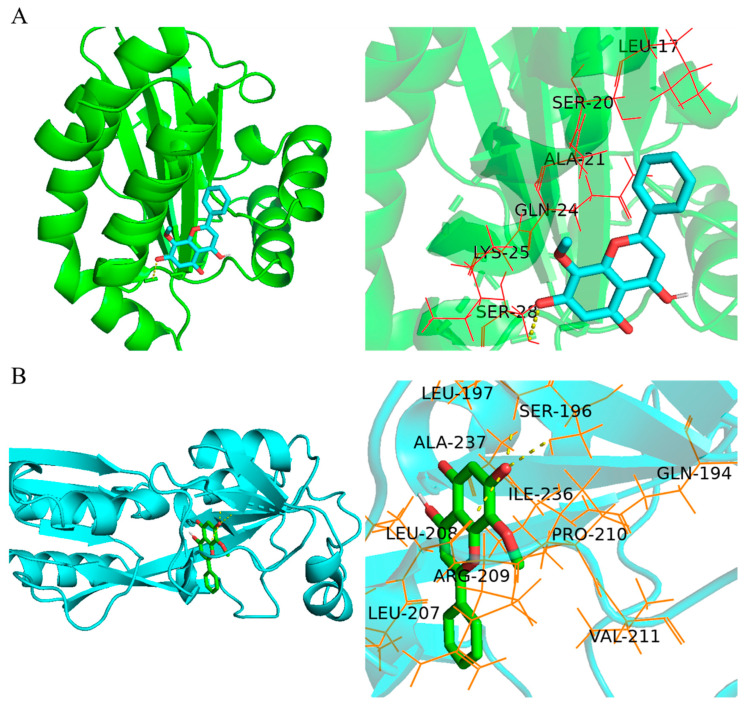
In silico molecular docking analysis. (**A**) The active sites of LasR CBD (PDBID:6D6A) docked with wogonin, including Ala, Ser, Gln, Leu, Lys, and Ser, and the binding energy is −2.44 kcal/mol. (**B**) The active sites of PqsR CBD (PDBID: 4JVI) docked with wogonin, including Ala, Arg, Ile, Pro, Val, and Gln, and the binding energy is −4.49 kcal/mol. 3D and 3D partial enlarged graphics were exhibited from left to right. Molecular docking was performed using Autodock Vina v.1.1.2, and graphics were generated with PyMOL v2.4.1 software.

In order to investigate the molecular mechanisms of wogonin against the QS systems of *P. aeruginosa* PAO1, molecular docking analysis in silico was performed to predict the targets of wogonin. The results showed that wogonin could bind LasR protein mainly with Ala, Ser, Gln, Leu, and Lys amino acids, and it also bound Ser by a hydrogen bond (Figure 5A and Figure S3A). The whole energy binding with LasR was −2.44 kcal/mol. Wogonin could bind PqsR protein mainly with Ala, Arg, Ile, Pro, Val, and Gln amino acids, and it also bound Ser and Leu by three hydrogen bonds (Figure 5B and Figure S3B). The whole energy binding with PqsR was −4.49 kcal/mol. As the 3D structure of RhlR was not available in the PDB database, the molecular docking analysis of RhlR was not performed. Taken together, it was concluded that wogonin could bind LasR and PqsR, and thereafter weakened the downstream virulence and pathogenic factors.

**Figure 6:**
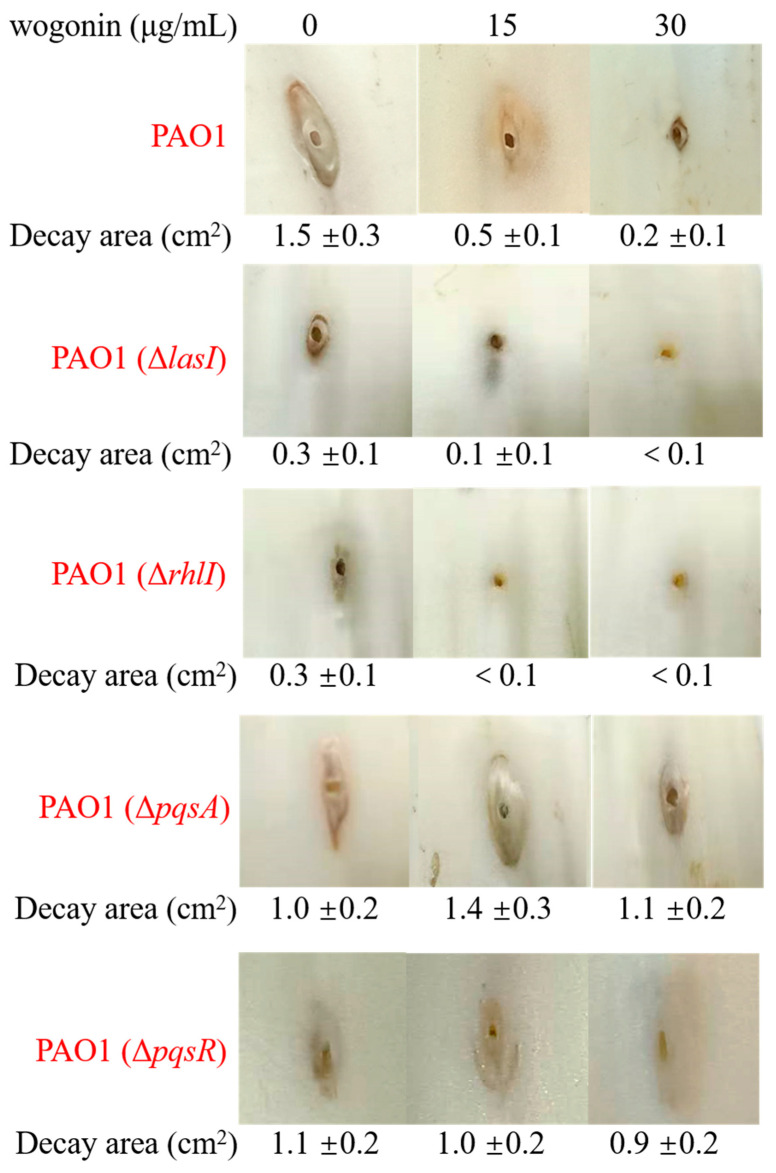
The decay area of cabbage after infection by various QS deletion mutants without or with 15 and 30 μg/mL wogonin, including PAO1 wild type strain, PAO1(Δ*lasI*), PAO1(Δ*rhlI*), PAO1(Δ*pqsA*), and PAO1(Δ*pqsR*). The errors represent standard errors.

**Figure 7:**
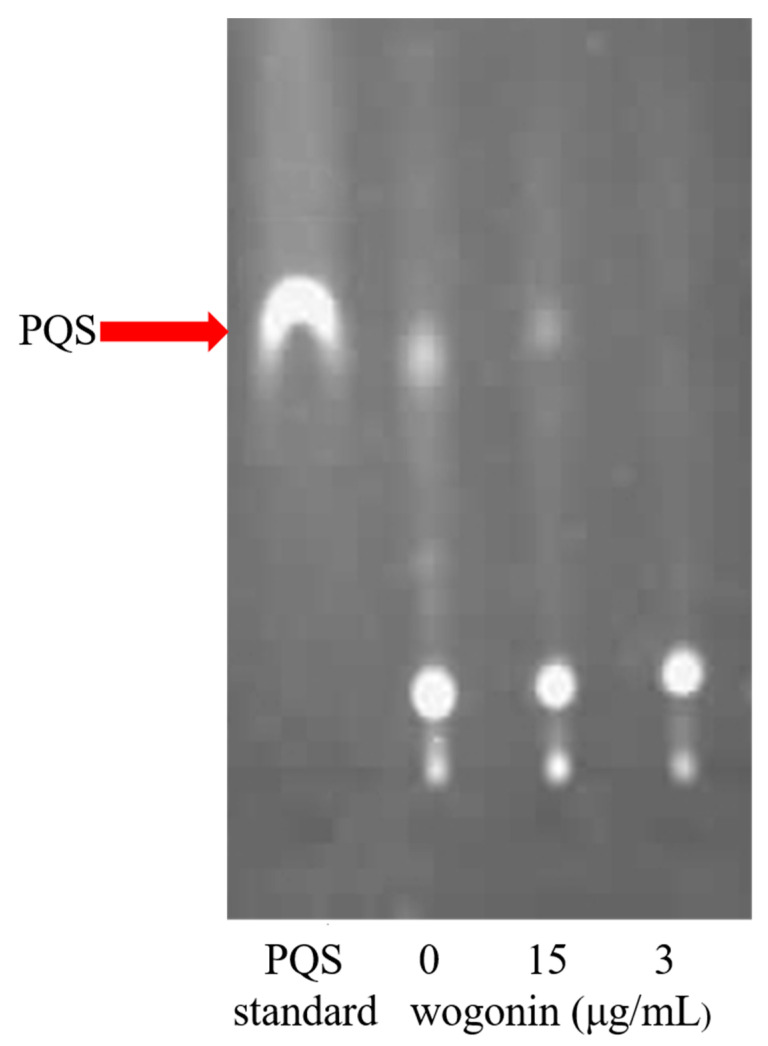
Wogonin inhibited PQS molecule production by TLC analysis. After being grown for 24 h without or with 15 and 30 μg/mL wogonin application, the PQS molecule from 5 mL culture of PAO1 was extracted and examined by TLC plate and photographed with long-wave UV light. Lane 1, PQS molecule standard; lane 2, no wogonin addition; lane 3, 15 μg/mL wogonin; lane 4, 30 μg/mL wogonin. The photograph was representative of three individual experiments.

The molecular docking results showed that wogonin could bind LasR and PqsR, but it was not clear which one took a dominant role. In order to identify the main target, we further examined the effects of wogonin on the pathogenicity of three QS-deleted mutants by the cabbage infection model. The results showed that wogonin attenuated the pathogenicity of the wild type strain, *lasI* deletion mutant, and *rhlI* deletion mutant, but not *pqsA* or *pqsR* single-gene deletion mutants, indicating that the *pqs* system played the dominant role during wogonin treatment (Figure 6). Furthermore, the TLC analysis for PQS production showed that wogonin at 15 μg/mL reduced the production of PQS signal molecules, and 30 μg/mL wogonin blocked the synthesis of PQS signal molecules (Figure 7). Taken together, all the results from the molecular docking and the pathogenicity analysis indicated that wogonin might affect PQS signal molecule production by interfering with *pqsA* or *pqsR*.

## 3. Discussion

*P. aeruginosa* is among the top three major human nosocomial pathogens to cause morbidity and mortality in immunocompromised patients. It often causes severe hospital-acquired infections, leading to bacteremia, pneumonia, and so on [3,25]. Virulence factors play important roles in the acute infection of *P. aeruginosa*. It is believed that anti-virulence therapy leads lower occurrence of drug resistance as it does not affect growth and exerts low selective pressure [26,27]. This bacterium can also form biofilms to cause chronic infection and increase bacterial drug resistance. Preventing biofilm formation or dispersing old biofilms is helpful in eradicating the infecting bacteria [5]. Most of the virulence factor production and biofilm formation are regulated by QS systems of *P. aeruginosa* [7]. Therefore, targeting QS systems may represent an adjunctive treatment approach for alleviating *P. aeruginosa* infection.

Many QSIs were developed to treat bacterial infections. Several natural extracts from food with plant origins have been found to display inhibition function for *P. aeruginosa* QS systems. For example, sulforaphane from broccoli and gingerol from ginger could inhibit the *las* system [27], vanillin impaired the *pqs* system [28], hordenine from sprouting barley downregulated three QS systems [29], leaf extracts of mango suppressed virulence factors and biofilm formation in the tested bacteria with unknown mechanisms [30], and pyranoanthocyanins from red wine interfered with QS system at distinct stages in a strain-specific manner [31]. Diallyl sulfide from garlic inhibited QS systems of *P. aeruginosa* and enhanced biosynthesis of three B vitamins through its thioether group [32].

Due to the abundance and pharmacological effects of traditional Chinese medicinal herbs, searching QSIs from traditional Chinese medicinal herbs may be promising. Among them, some have been found to show anti-QS function. For example, the extracts from the mixture of *Artemisiae argyi* folium, the root bark of *Cortex dictamni*, and the root of *Solanum melongena* could completely inhibit *pqs* system of *P. aeruginosa* [21]. Paeonol from peony root interfered with *las* system, reduced virulence production, and inhibited biofilm formation [23]. Coumarin, a Chinese medicinal herb-derived phenolic compound, reduced protease and pyocyanin production and inhibited biofilm formation of different *P. aeruginosa* strains by impairing QS systems and changing cyclic diguanylate (c-di-GMP) metabolism [33]. Okanin in *Coreopsis tinctoria* Nutt interfered with QS system of *Chromobacterium violaceum* by the downregulation of *vio* operon [22].

In this study, wogonin, the active ingredient from *A. pilosa*, was found to target the *pqs* QS system of *P. aeruginosa* to inhibit PQS molecule synthesis. Consequently, wogonin reduced the production of many virulence factors and weakened the pathogenicity of the bacterium. It could effectively protect plant stem from this bacterial infection and increase the survival rates of fruit fly and nematode after infection. Taken together, wogonin attenuated the pathogenicity of *P. aeruginosa* both in acute and chronic infections, indicating that wogonin could be an alternative method to inhibit human opportunistic pathogen *P. aeruginosa*. However, in order to broaden its application scope, it is worthy of further investigation to verify its function for treating infections due to other human pathogens. It is also interesting to use antibiotics together with QSIs to synergistically treat bacterial infections in the future.

It is worth mentioning that although traditional Chinese medicinal herbs with the function of “Qing Re Jie Du” have been used to treat many common diseases in China for thousands of years, some underlying mechanisms remain unknown. *A. pilosa* has been found to have the effects of anti-inflammation, anti-oxidation, and anti-tumor, and is widely used for the treatment of diabetes, tumor, Meniere’s syndrome, trichomonas vaginitis, and other diseases [34]. It was reported that the active components of *A. pilosa* contain flavonoids, triterpenoids, tannins, phenols, and other chemical substances. Wogonin, an ingredient of *A. pilosa*, belongs to flavonoids and has been used as an inhibitor of inflammatory mediators produced by macrophages, lymphocytes, microglia, and endothelial cells [24]. The molecular mechanisms of inhibition involve several signaling pathways, such as ER stress-mediated apoptosis and autophagy, mitogen-activated protein kinase (MAPK), and transcription factor inhibition of NF-κB [35]. Here, it was found that the crude extracts of *A. pilosa* and an ingredient wogonin both could inhibit most of virulence factors and biofilm formation to attenuate pathogenicity of *P. aeruginosa* PAO1 by targeting *pqs* system. These results help to explain the antibacterial activity of traditional Chinese medicinal herbs with the function of “Qing Re Jie Du”. It also provides a research direction to examine the effects of other traditional Chinese medicinal herbs with similar function.

## 4. Materials and Methods

### 4.1. Bacterial Strains, Plasmids and Culture Conditions

Bacterial strains used in the study are shown in Table 2. LB (Luria–Bertani) broth and agar were used for bacterial culture at 37 °C. When necessary, the media were supplemented with kanamycin (Kan, 50 μg/mL) or trimethoprim (Tmp, 300 μg/mL) purchased from Amresco (Solon, PA, USA). 

### 4.2. Active Substance Extraction of Traditional Chinese Medicinal Herbs

Eighteen kinds of traditional Chinese medicinal herbs with the function of “Qing Re Jie Du” were purchased from the local pharmacy (Tongrentang, Beijing, China). For extraction of active substances, the grounded herb was first immersed in deionized water or 70% ethanol, respectively. For deionized water extraction, the herb was boiled for 2 h twice and the extracted liquids were merged. Regarding the 70% ethanol extraction, the heat reflux method was used. Then, the two kinds of extracts were separately filtered by Buchi funnel, concentrated by a rotary evaporator, freeze-dried by a vacuum freeze dryer, and kept at −20 °C. Before the examination, extracts were, respectively, dissolved in deionized water (it was designed as the water extracts) or 70% ethanol (it was designed as the ethanol extracts) and sterilized using a 0.22 μm filter. Wogonin standard was purchased from Shanghai Yuanye Bio-Technology Co. Ltd. (Shanghai, China).

### 4.3. Gene Expression Assay

The expression levels of the QS-related genes including *lasI, lasR, rhlI, rhlR, lasB,* and *rhlA* were analyzed using the *lux-*reporter system as our previous study [46]. In brief, the promoter regions of these target genes were, respectively, fused with the *luxCDABE* genes in the vector pMS402. The strains containing the different promoters-*luxCDABE* fusion reporters were inoculated in LB broth supplemented with 300 μg/mL Tmp overnight. Then, the culture was transferred to the new LB broth at a ratio of 1:100 and incubated for ~3 h until the log-growth phase. Five microliters of log-phase culture was inoculated into parallel wells on a 96-well black plate containing 95 μL of LB broth with the water or ethanol extracts of different Chinese medicinal herbs and wogonin, respectively. To prevent evaporation, 60 μL of mineral oil was added into each well. Promoter activity and bacterial growth were measured every 30 min for 24 h in a Victor multilabel plate reader (Wallac model 1450, Perkin-Elmer, MA, USA).

### 4.4. Measurement of Virulence Factors

#### 4.4.1. Pyocyanin

Pyocyanin was analyzed according to the previous report [47]. Briefly, after growing in *Pseudomonas* broth medium (0.14%NaCl, 1%K_2_SO_4_, and 2% tryptone) with different concentrations of the extract of *A. pilosa* or wogonin at 37 °C for 24 h, the culture was centrifuged to obtain supernatant. After 3 mL of chloroform was added to 5 mL of supernatant and vigorously vortexed for 2 min, the mixture was centrifuged at 8000 rpm for 10 min. Then, the chloroform layer was transferred to a fresh tube and mixed with 1 mL of 0.2 M HCl. After centrifugation again, the top layer was removed and OD_520_ was measured. The amount of pyocyanin (μg/mL) was obtained using the formula: OD_520_/OD_600_ × 17.072.

#### 4.4.2. Biofilm Formation

The biofilm formation of wild type strain PAO1 with or without the extracts of *A. pilosa* or wogonin was tested according to the previous method [48]. The strain with different concentrations of the extracts of *A. pilosa* or wogonin was added in sterile test tubes and incubated at 37 °C until the biofilm was formed. Followed by removal of non-adherent cells from the tubes and gentle rinsing three times with sterile PBS, biofilm biomass was evaluated by crystal violet staining. The attached crystal violet was washed with anhydrous ethanol and its absorbance was measured at OD_570_ and analyzed.

In order to determine the effects of wogonin on the formed biofilm, after PAO1 was grown in a 96-well polyvinyl chloride plate (Corning/Costar, Corning, NY, USA) at 37 °C for 10 h, the culture was removed and new LB broth with 15 or 30 μg/mL wogonin was added and incubated at 37 °C overnight. The biofilm biomass was evaluated by crystal violet staining as the above-mentioned. Each samples had 12 replicates.

#### 4.4.3. Proteolytic Enzyme

After diluting overnight culture to OD_600_ = 0.2, 2 μL of aliquots were spotted onto LB agar with 5% skim milk with or without wogonin addition. After incubation at 37 °C overnight, the hydrolysis ring diameters were determined.

#### 4.4.4. Motility

The motility was determined in three kinds of media as previously reported [49]. The medium used for swarming motility assay consisted of 0.6% agar, 0.8% nutrient broth, and 0.5% glucose with or without the extracts of *A. pilosa* or wogonin. The medium for swimming motility assay consisted of 0.3% agar, 0.5% NaCl, and 1% peptone with or without the extracts of *A. pilosa* or wogonin. The medium used for twitching motility assay was thin LB agar plates with or without the extracts of *A. pilosa* or wogonin. All the plates were dried at room temperature overnight before use. Two microliters of diluted overnight culture (OD_600_ = 0.5) was spotted on swarming or swimming agar plates. After the samples were incubated at 37 °C for 24 h, the swarming and swimming motility diameters were measured. The twitching motility was observed by stab-inoculating bacteria through the thin LB medium. After incubated at 37 °C for 48 h, the twitching bacterial cells were stained with crystal violet and washed with PBS to determine diameters.

#### 4.4.5. Elastase

Elastase assay was performed as previously described [50]. In brief, PAO1 was grown in LB broth with the addition of different concentrations of the extracts of *A. pilosa*. Then, 1 mL of overnight culture was centrifuged, and the supernatant was kept. After the supernatant was added into a new tube with 10 mg/mL Elastin-Congo Red (Sigma, Saint Louis, MO, USA) in 2 mL of reaction buffer (1 mM CaCl_2_, 0.1 M Tris-HCl buffer, pH 7.0) and kept at 37 °C for 3 h, the mixture was centrifuged at 3000× *g* for 10 min. OD_495_ of the supernatant was used to estimate elastase activity.

#### 4.4.6. Exopolysaccharide Production Assay

Psl polysaccharide production assay was carried out according to the previous method [51]. In brief, the diluted overnight of PAO1 (OD_600_ = 0.005) was spotted onto Congo Red plates (1% tryptone, 1% agar, 4% Congo Red, and 2% Coomassie blue) with or without wogonin. The colony morphology and color were observed after 3 d incubation at 37 °C.

#### 4.4.7. Rhamnolipid Measurement

The rhamnolipid was measured as previously described [49]. In brief, after being grown in LB broth with or without the extracts of *A. pilosa* for 2 d, the culture was centrifuged at 12,000 rpm for 5 min. The supernatant was mixed with an equal volume of ethyl acetate and vortexed vigorously. After centrifugation, the organic phase was collected and dissolved in 4 mL of chloroform and added in 200 μL of freshly prepared 1 g/L methylene blue solution (pH 8.6). After vortexing for 4 min and standing for 15 min, OD_638_ of the solution was measured, and the values were calculated to obtain rhamnolipid concentration according to a calibration curve.

### 4.5. In Vivo Pathogenicity Assay

The pathogenicity of PAO1 with or without wogonin application was investigated using Chinese cabbage (*B. pekinensis*), fruit fly (*D. melanogaster* Canton S), and nematode (*C. elegans* N2) infection models as reported previously [52,53,54].

For the Chinese cabbage infection assay, the overnight culture was centrifuged and resuspended to OD_600_ = 2.0 in 10 mM MgSO_4_. After the surface of Chinese cabbage was disinfected with 0.1% H_2_O_2_, ten microliters of bacterial cells with or without the extracts of *A. pilosa* or wogonin was inoculated on the stem of Chinese cabbage for 6 d at 30 °C and the rot area of Chinese cabbage was observed and analyzed.

For the fruit fly infection assay, the cells from 1.0 mL of the overnight culture were collected by centrifugation and resuspended in 5% sucrose with an OD_600_ of 2.0. Then, 100 μL of the resuspended cells with or without wogonin was spotted onto a sterile filter that was placed on the surface of 2 mL of solidified agar with 5% sucrose in a 20 mL glass tube. Before adding to each tube, all the fruit flies were starved for 3 h. The cold shock method was used to anesthetize fruit flies during sorting and transferring. The tubes with fruit flies were stored at 25 °C in a humidity-controlled environment. The number of live fruit flies was counted and documented at 24 h intervals for 12 d.

For the nematode infection examination, NGM plates (0.3% NaCl, 0.25% tryptone, 1 mM MgSO_4_, 1 mM CaCl_2_, 5 μg/mL cholesterol, 100 μg/mL FUDR, and 2% agar) with or without wogonin was used. The nematodes in the L4 phase by synchronization treatment were transferred to the NGM plates, and then the survival rate of nematodes was recorded every 12 h for 5 d.

### 4.6. Molecular Dynamics Simulation

Molecular dynamics simulation was carried out according to the previous report [55]. In brief, wogonin was used to dock the transcriptional regulators of QS circuits of *P. aeruginosa* PAO1, LasR, and PqsR. Due to the lack of crystal structure, RhlR was not analyzed. The 3D structure of the two proteins—LasR (PDB ID: 6D6A) and PqsR (PDB ID: 4JVI)—was downloaded from Protein Data Bank (http://www.pdb.org, accessed on 30 September 2021). The 3D-crystal structure of wogonin was docked to the binding sites of LasR and PqsR using Auto Dock Vina. The optimal conformation was confirmed based on the number of hydrogen bonds, hydrophobic interaction force, and binding energy. The 2D-crystal structures and 3D-crystal structures were, respectively, obtained by Discovery Studio2016 and PyMOL v2.4.1 software.

### 4.7. PQS Signal Molecule Production Assay

PQS production was analyzed as previously described [56]. Briefly, after growth for 24 h, a 5 mL culture of PAO1 was collected and extracted three times with acidified ethyl acetate. The extract was dried and resuspended in the mixture with ethyl acetate and acetonitrile (*v*/*v*, 1:1). One microliter extract was loaded onto a TLC plate. Dichloromethane and methanol (*v*/*v*, 95:5) were used as the developing solvent. After the components of the samples were separated on TLC plates, the plates were photographed with long-wave UV light.

### 4.8. Construction and Complementation of In-Frame Deletion Mutants

The in-frame deletion mutation was performed by a *sacB*-based method [40]. In brief, the upstream and downstream fragments of the gene *pqsA* were amplified by the primers sets *pqsA*-up1, *pqsA*-down1, *pqsA*-up2, and *pqsA*-down2 (Table 3). After the fragments were ligated with the vector pEX18Tc by overlapping sequences, the plasmid pEX18Tc-*pqsA* was consequently obtained. The *pqsA* gene deletion was carried out by triparental mating as previously described [57]. Briefly, PAO1 and *E. coli* DH10B strains containing pEX18Tc-*pqsA* or pRK2013 were, respectively, grown overnight, collected, washed with PBS, and suspended in SOC medium. The bacterial mixture with a ratio of 1:1:1 was spotted onto a LB agar plate. After growth at 37 °C for 12 h, the bacterial cells were resuspended in 500 µL of LB liquid medium and plated onto PIA plates with 300 μg/mL tetracycline addition. The obtained mutant was verified by PCR.

To complement *pqsA* deletion, after the gene *pqsA* was amplified using the primer sets pAK-*pqsA*-up and pAK-*pqsA*-down (Table 3) and ligated into pAK1900 vector, the vector was transferred into *E. coli* DH10B by electroporation. Integrants were selected on PIA plates containing 250 μg/mL Cb.

### 4.9. Statistical Analysis

Statistical analysis was performed using GraphPad Prism version 5.0.1. All the experiments were repeated at least three times. One-way analysis of variance (ANOVA) was performed to determine significant differences (*p* < 0.05). 

## 5. Conclusions

The anti-QS activity from the extracts of 18 traditional Chinese medicinal herbs with the function of “Qing Re Jie Du” was examined. Among them, the crude extracts of *A. pilosa* showed the optimal inhibitory effects against QS systems. The extracts weakened most of virulence factors and pathogenicity of *P. aeruginosa* PAO1, inhibited biofilm formation, but did not influence this bacterial growth under the condition with the tested concentrations. Further investigation showed that wogonin, one of the active ingredients of *A. pilosa*, exhibited similar phenotypes with the crude extracts of *A. pilosa*. It downregulated the QS-related gene expression and attenuated most of the virulence factor production and bacterial pathogenicity in cabbage infection and fruit fly and nematode survival experiments. In silico molecular docking analysis, the pathogenicity observation of various QS-related mutants and PQS signal molecule examination confirmed that wogonin could mainly interfere with PQS signal molecular synthesis by affecting *pqsA* and *pqsR*. These results are helpful for understanding the function of traditional Chinese medicinal herbs named “Qing Re Jie Du” and providing an alternative approach for bacterial infections.

## Figures and Tables

**Table 1:** Crude extract concentrations of different Chinese medicinal herbs and the effects on QS-related genes.

Chinese Medicinal Herbs	Extraction Solvent ^a^	Concentration (mg/mL)	*lasR* ^b^	*lasI*	*rhlR*	*rhlI*	*pqsH*	*phzM*	*phzA1*	*phzA2*
*Agrimonia pilosa*	water	56	↓	↓	↓	-	↑	-	↓	↓
	ethanol	27	↓	↓	↓	-	-	↓	-	↓
*Sargentodoxa cuneata*	water	88	-	-	↓	-	-	↓	-	-
	ethanol	34	-	-	↑	-	↓	↓	↓	-
*Ash bark*	water	60	-	-	↓	-	↓	↑	↓	-
	ethanol	13.4	-	-	-	-	↓	-	-	-
*Rhizoma paridis*	water	78	↑	↑	↑	-	-	↑	-	-
	ethanol	47	-	-	-	-	-	↓	-	-
*Herba asari*	water	182	-	-	↑	↑	↑	↑	↑	↑
	ethanol	17.5	-	-	-	-	↓	-	-	-
*Stephania tetrandra*	water	40	-	-	-	-	-	-	-	-
	ethanol	14	-	-	-	-	-	-	-	-
*Lonicerae japonicae*	water	108	-	↑	↑	-	-	↑	-	↑
	ethanol	36	-	-	-	-	-	-	↓	-
*Tussilago farfart*	water	202	-	-	-	-	-	-	↓	-
	ethanol	44	-	-	-	-	-	-	↓	-
*Isatidids folium*	water	350	-	-	↑	↑	↑	↑	↑	↑
	ethanol	34	-	-	-	-	-	-	-	-
*Balloon flower*	water	330	-	↓	↑	↑	↑	↑	↑	↑
	ethanol	52	-	-	-	-	-	-	-	↓
*Radix arnebiae*	water	10	↓	-	-	↓	-	↓	-	-
	ethanol	10	-	-	-	-	-	-	-	-
*Hedyotis diffusa*	water	104	-	-	-	-	-	-	-	-
	ethanol	12.5	-	-	-	-	-	-	-	-
*Herba lobeliae*	water	186	↑	↑	↑	-	↑	-	-	-
	ethanol	18	-	-	-	-	-	-	-	↓
*Dwarf lilyturf*	water	396	-	-	↑	↑	↑	↑	↑	↑
	ethanol	50	-	-	-	-	-	↓	-	-
*Pulsa tilia radix*	water	68	-	-	-	↓	↓	↓	-	-
	ethanol	40	-	-	-	-	-	-	-	-
*Subprostrate sophra*	water	106	-	↑	↑	-	↑	-	-	-
	ethanol	35	-	-	-	-	-	↓	↓	-
*Asteris rasix*	water	288	↑	↑	-	-	↓	↓	-	-
	ethanol	73	-	↓	-	-	-	-	↓	↓
*Cortex dictamni*	water	170	-	-	↑	↑	↑	↑	↑	↑
	ethanol	34	-	-	↑	-	↓	-	-	-

^a^ Chinese medicinal herbs were extracted by deionized water and 70% ethanol, respectively. ^b^ up arrows mean increased expression; down arrows mean reduced expression; transverse lines mean no change.

**Table 2:** Bacterial strains used in this study.

Strains/Plasmids	Description	Source or Reference
Strains		
*E. coli* DH10B	*F^–^ mcrA* Δ*(mrr^-^hsdRMS^-^mcrBC)* φ*80dlacZ* Δ*M15* Δ*lacX74 recA1 ara*Δ*139* Δ*(ara leu)7697 galU galK rpsL* (Str^R^) *endA1 nupG*	Invitrogen
PAO1	*P. aeruginosa* wild type strain	This lab
PAO1 (Δ*lasI*)	*lasI* deletion mutant of PAO1	[36]
PAO1 (Δ*rhlI*)	*rhlI* deletion mutant of PAO1	[37]
PAO1 (Δ*pqsA*)	*pqsA* deletion mutant of PAO1	This study
PAO1 (Δ*pqsR)*	*pqsR* deletion mutant of PAO1	[38]
Δ*pqsA*/CTX-*pqsA*	CTX-*pqsA* integrated into PAO1(Δ*pqsA*) chromosome; Tc^r^	This study
Δ*pqsA*/pAK-*pqsA*/CTX-*pqsA*	Δ*pqsA*/CTX-*pqsA* complemented by plasmid pAK-*pqsA*; Cb^r^	This study
Δ*pqsR*/CTX-*pqsA*	CTX-*pqsA* integrated into PAO1(Δ*pqsR*) chromosome; Tc^r^	This study
Δ*pqsR*/pAK-*pqsR*/CTX-*pqsA*	Δ*pqsR*/CTX-*pqsA* complemented by plasmid pAK-*pqsR*; Cb^r^	This study
Plasmids		
pMS402	Expression reporter vector carrying the promoterless *luxCDABE*; Kan^r^, Tmp^r^	[39]
pEX18-Tc	Broad host range gene replacement vector; *sacB*, Tc^r^	[40]
pRK2013	Broad host range helper vector; Traþ, Kan^r^	[41]
pKD-*lasI*	pMS402-containing *lasI* promoter region; Kan^r^, Tmp^r^	[42]
pKD-*lasR*	pMS402-containing *lasR* promoter region; Kan^r^, Tmp^r^	[42]
pKD-*lasB*	pMS402-containing *lasB* promoter region; Kan^r^, Tmp^r^	This lab
pKD-*rhlI*	pMS402-containing *rhlI* promoter region; Kan^r^, Tmp^r^	[42]
pKD-*rhlR*	pMS402-containing *rhlR* promoter region; Kan^r^, Tmp^r^	[42]
pKD-*rhlA*	pMS402-containing *rhlA* promoter region; Kan^r^, Tmp^r^	This lab
pKD-*pqsA*	pMS402-containing *pqsA* promoter region; Kan^r^, Tmp^r^	[43]
pKD-*pqsR*	pMS402-containing *pqsR* promoter region; Kan^r^, Tmp^r^	[43]
pKD-*flgB*	pMS402-containing *flgB* promoter region; Kan^r^, Tmp^r^	This lab
mini-CTX-*lacZ*	Integration plasmid-containing *attP* site for integration at chromosomal *attB* site; Tc^r^	[44]
CTX-*pqsA*	mini-CTX-*lacZ*-containing *pqsA* gene region	This study
pAK1900	*E. coli-P. aeruginosa* shuttle cloning vector carrying p*_lac_* upstream of MCS; Amp^r^, Cb^r^	[45]
pAK-*pqsA*	pAK1900-containing *pqsA* gene region	This study
pAK-*pqsR*	pAK1900-containing *pqsR* gene region	This study

**Table 3:** Primers used in this study.

Primers	Sequence (5′→3′)	Restriction Sites	Function
pKD-*pqsA*-up	CATCTCGAGCCAGGGTCAGTGTCC	*Xho* I	Luminous reporter construction
pKD-*pqsA*-down	TGTGGATCCCAGAACCTCGGTCAG	*BamH* I	Luminous reporter construction
*pqsA*-up1	CAGGAAACAGCTATGACCATGATTACGAAGTACCTTGGAAGTCGAGC	*-*	*pqsA* knockout
*pqsA*-down1	CAACATGCCCGTTCCTCGGAACAGAACCTCGGTC	*-*	*pqsA* knockout
*pqsA*-up 2	GACCGAGGTTCTGTTCCGAGGAACGGGCATGTTG	-	*pqsA* knockout
*pqsA*-down 2	CGACGGCCAGTGCCAAGCTTCGAACGGATCGAAGCTG	-	*pqsA* knockout
*pAK-pqsA*-up	TATGAGCTCCCGTGGTTCTTCTCC	*Sac* I	*pqsA* complement
*pAK-pqsA*-down	CGTTCTAGAGCATCAGCATCTCGTC	*Xba* I	*pqsA* complement
*pAK-pqsR*-up	TATTTAGGTGACACTATAGAATACTCAAGCTTGAATAAGGGATGCCTATTC	*Hind* III	*pqsR* complement
*pAK-pqsR*-down	GAATTGTAATACGACTCACTATAGGGCGAATTCGAGCTCCCACGGCCAGCGTC	*EcoR* I	*pqsR* complement

## Supplementary Materials

The following are available online at https://www.mdpi.com/article/10.3390/ijms222312699/s1.
