# Supplementary material for: Inhibition of Virulence Factors and Biofilm Formation by Wogonin Attenuates Pathogenicity of Pseudomonas aeruginosa PAO1 via Targeting pqs Quorum-Sensing System
Source: Int J Mol Sci. 2021 Nov 24;22(23):12699. doi: 10.3390/ijms222312699 (PMC8657757; doi:10.3390/ijms222312699)
Supplement: Supplementary file 1 [file ijms-22-12699-s001.zip › ijms-1423027-supplementary.pdf]

Supplementary Materials

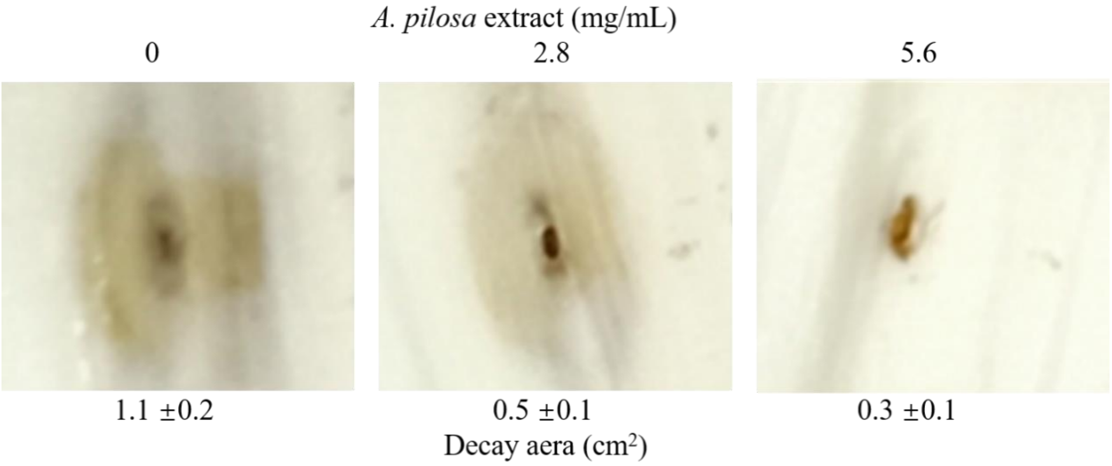

Figure S1. The decay area of Chinese cabbage after infection by various concentrations of the extracts from *A. pilosa*. The errors are standard errors.

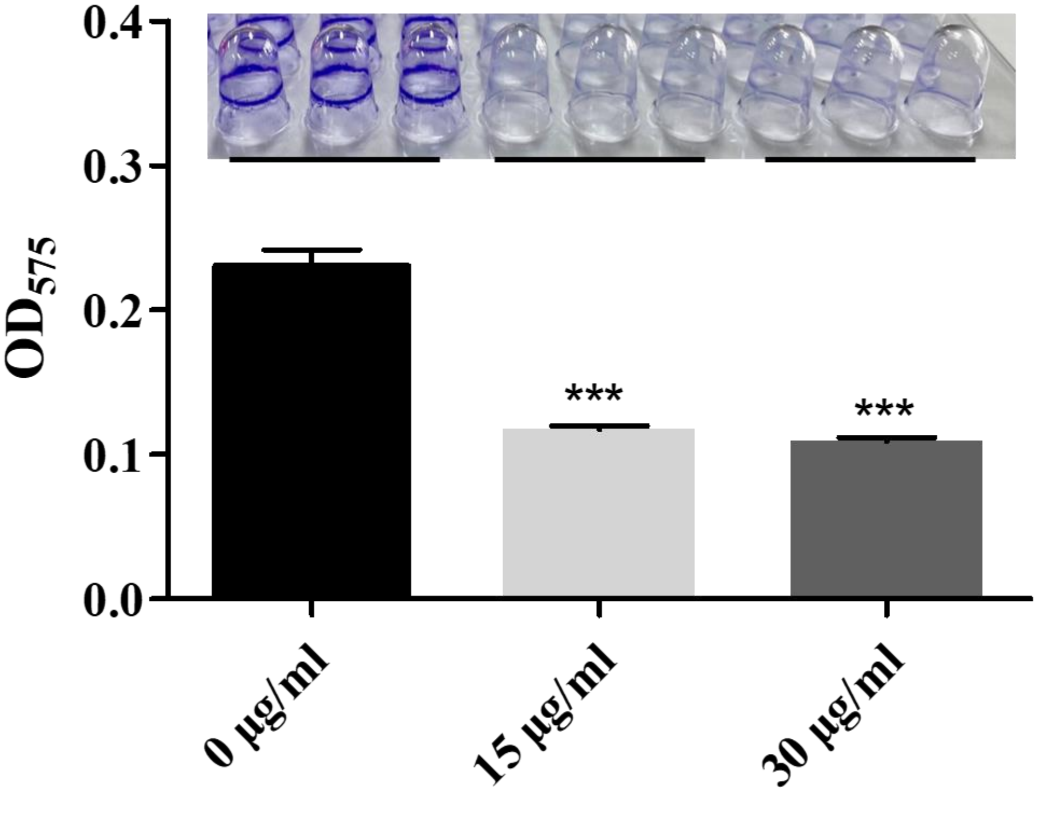

Figure S2. The effects of wogonin on the formed biofilm. After PAO1 was grown in a 96-well plate at 37 °C for 10 h, the culture was removed and new LB broth with 15 or 30 µg/mL wogonin was added and incubated at 37 °C overnight. The biofilm biomass was evaluated by crystal violet staining. Each sample displayed 3 wells. The errors are standard errors. The error bars represent standard errors. ***, $p < 0.001$.

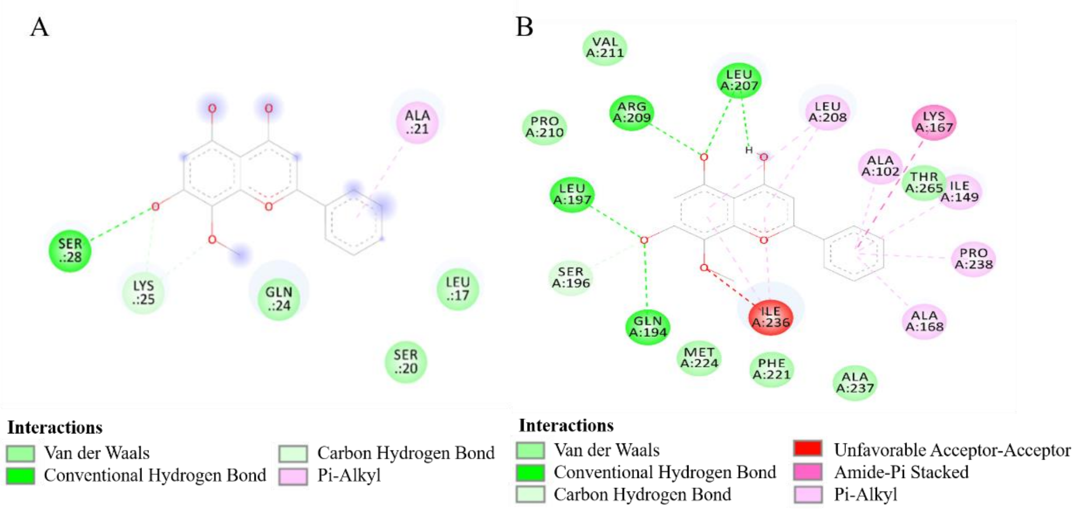

Figure S3. In silico molecular docking analysis. (**A**) The active sites of LasR CBD (PDBID:6D6A) docked with wogonin. (**B**) The active sites of PqsR CBD (PDBID: 4JVI) with wogonin. Molecular docking was performed using Autodock Vina v.1.1.2, and graphics were generated with Discovery Studio2016 software.
